# Dynamic cortical participation during bilateral, cyclical ankle movements: Effects of Parkinson’s disease

**DOI:** 10.1371/journal.pone.0196177

**Published:** 2018-04-26

**Authors:** Takashi Yoshida, Kei Masani, Karl Zabjek, Milos R. Popovic, Robert Chen

**Affiliations:** 1 Rehabilitation Engineering Laboratory, Toronto Rehabilitation Institute, University Health Network, Toronto, Ontario, Canada; 2 Institute of Biomaterials and Biomedical Engineering, University of Toronto, Toronto, Ontario, Canada; 3 Applied Surgical and Rehabilitation Technology Lab, Department of Trauma Surgery, Orthopaedics and Plastic Surgery, University Medical Center Göttingen, Göttingen, Lower Saxony, Germany; 4 Department of Physical Therapy, University of Toronto, Toronto, Ontario, Canada; 5 Division of Neurology, Department of Medicine, University of Toronto, Toronto, Ontario, Canada; 6 Krembil Research Institute, University Health Network, Toronto, Ontario, Canada; 7 Edmond J. Safra Program in Parkinson’s Disease, University Health Network, Toronto, Ontario, Canada; Tokai University, JAPAN

## Abstract

Parkinson’s disease (PD) is known to increase asymmetry and variability of bilateral movements. However, the mechanisms of such abnormalities are not fully understood. Here, we aimed to investigate whether kinematic abnormalities are related to cortical participation during bilateral, cyclical ankle movements, which required *i*) maintenance of a specific frequency and *ii*) bilateral coordination of the lower limbs in an anti-phasic manner. We analyzed electroencephalographic and electromyographic signals from nine men with PD and nine aged-matched healthy men while they sat and cyclically dorsi- and plantarflexed their feet. This movement was performed at a similar cadence to normal walking under two conditions: *i*) self-paced and *ii*) externally paced by a metronome. Participants with PD exhibited reduced range of motion and more variable bilateral coordination. However, participants with and without PD did not differ in the magnitude of corticomuscular coherence between the midline cortical areas and tibialis anterior and medial gastrocnemius muscles. This finding suggests that either the kinematic abnormalities were related to processes outside linear corticomuscular communication or PD-related changes in neural correlates maintained corticomuscular communication but not motor performance.

## Introduction

Parkinson’s disease (PD) is known to increase the variability and asymmetry of bilateral rhythmical movements, such as walking [1–8]. Although the hallmark of PD is well established as progressive neuronal degeneration, mechanisms of many specific kinematic abnormalities in PD are not clearly understood.

Corticomuscular coherence in the beta band had been used to suggest that synchronous cortical oscillations were functionally related to muscle activities during sustained contractions about single joints [9,10]. In our previous studies, we demonstrated that the coherence between the primary sensorimotor cortex and the active leg muscles increased cyclically in the beta band (13 to 30 Hz) during bilateral, cyclical ankle movements [11,12]. Because the ankle movements that we adopted had only a few intended functional requirements such as maintaining a specific movement frequency and coordinating the feet in an anti-phasic manner, our findings suggest that the primary sensorimotor cortex may contribute to these requirements via corticomuscular communication [11,12].

In addition to the aforementioned effects on the consistency and symmetry of bilateral movements [1–8], PD can impair the performance of anti-phasic movements [13–19]. Because these kinematic features are fundamental to the aforementioned ankle movements, it is likely that individuals with PD will perform these movements abnormally. Furthermore, if the cyclical corticomuscular coherence during the ankle movements is relevant to specific functional requirements, kinematic abnormalities in PD would be accompanied by corresponding changes in corticomuscular coherence.

Only a few studies have examined how PD affects corticomuscular coherence, and the experimental evidence is limited to sustained contractions of upper limb muscles [20,21]. During sustained isometric extension of the wrist, healthy individuals and individuals with PD on levodopa show similar magnitudes of coherence within the beta band [20]. In the off-medication condition, individuals with PD showed decreased beta coherence [20], suggesting that dopamine deficiency within the basal ganglia impairs corticomuscular synchronization. To our knowledge, no study has examined corticomuscular coherence during bilateral cyclical movements of the lower limbs at a cadence similar to normal walking in PD.

Although the corticospinal connection may be normal in PD, indicated by the preservation of central motor conduction time [22,23], PD may still affect corticomuscular communication. This is suggested by the anatomical and physiological relationship between the basal ganglia and motor cortex. The basal ganglia, which is affected by degeneration of dopaminergic neurons in PD [24–26], is reciprocally connected with the motor cortices [27–30]. In rat models of PD, the motor cortex is coherent with the substantia nigra pars reticulata during treadmill walking [31] and the subthalamic nucleus during sustained exploratory movement [32]. Furthermore, such cortico-basal ganglia coherence is abolished by L-dopa or dopamine receptor agonist [31,32] and restored by dopamine D_2_ receptor antagonist [31], suggesting that the coherence is pathological. Similar cortico-basal ganglia coherence and its dopamine dependence have been observed in individuals with PD during rest or tonic wrist extension [33,34]. If the basal ganglia and motor cortex interact abnormally during the ankle movements, then it is possible that changes in cortical activities may alter corticomuscular communication though the exact effects are uncertain.

Here, we aimed to investigate the mechanism of kinematic abnormalities in PD by examining the corticomuscular communication between the midline cortical areas and the active muscles during bilateral, cyclical ankle movements at a cadence similar to normal walking. To quantify the corticomuscular communication, we calculated the coherence between electroencephalographic (EEG) and electromyographic (EMG) signals. The experimental tasks were performed under two conditions (self-paced and externally-paced by a metronome) as rhythmic aural pacing, at or slightly faster than the preferred cadence, can acutely reduce movement variability in individuals with PD [4], and such change in motor performance may be accompanied by changes in corticomuscular coherence.

Based on the existing evidence, we hypothesized that, compared to healthy individuals, the magnitude of coherence between contracting muscles and the midline primary sensorimotor cortex within the beta band would be lower in individuals with PD. Participants were assessed in the off-medication condition because dopamine has been shown to restore corticomuscular coherence [20] and normalize the interaction between the basal ganglia and motor cortex [31,32].

## Materials and methods

### Participants

We recruited ten men with PD and eleven aged-matched healthy men. The same sample of healthy men was reported in our previous study [12]. One participant with PD and two healthy participants were excluded from data analysis because of excessive EEG artifacts. The remaining nine participants with PD were 62±7 years old and healthy participants were 66±7 years old (mean±standard deviation). The two groups of participants did not differ significantly in age (*p* = .227, unpaired *t*-test). The clinical details of the participants with PD are summarized in Table 1.

**Table 1:** Clinical details of participants with PD.

Age (years)	Disease Duration (years)	Medication (mg/day)	Predominant Motor Symptom	UPDRS III Score (out of 108)	MoCA Score (out of 30)	GFQ Score (out of 64)
63	15	Levodopa (400 mg); Carbidopa (100 mg); Rasagiline (1 mg); Pramipexole (4.5 mg)	Bradykinesia and tremor in right arm; reduced swing of right arm during walking; postural lean to left side	23	29	16
51	9	Levodopa (450 mg); Carbidopa (112.5 mg)	Resting tremor in left hand	19	28	N/A
64	9	Levodopa (800 mg); Carbidopa (200 mg); Amantadine (200 mg)	Resting tremor in left hand; wearing off; difficulty raising left leg during walking	11	28	N/A
67	9	Levodopa (600 mg); Carbidopa (150 mg)	Tremor in left hand; dystonia of upper and lower extremities; micrographia; occasional extension of first left toe; bradykinesia	18	29	N/A
62	5	Levodopa (300 mg); Carbidopa (75 mg); Rasagiline (1 mg); Pramipexole (2.25 mg)	Reduced swing of right arm during walking; bradykinesia and reduced dexterity of right hand; micrographia; dystonia of second right toe	6	27	N/A
62	15	Levodopa (400 mg); Carbidopa (100 mg); Ropinirole (6 mg)	left-sided tremor; generalized dyskinesia; impaired speech; wearing off	28	25	10
52	3	Levodopa (300 mg); Carbidopa (75 mg); Domperidone (30 mg)	Right-sided rigidity; right-sided resting and action tremor; reduced swing of right arm during walking	20	24	N/A
66	6	Levodopa (700 mg); Carbidopa (175 mg)	Right-sided bradykinesia and rigidity	20	27	12
71	20	Levodopa (1000 mg); Carbidopa (100 mg)	Rigidity; generalized bradykinesia	13	25	10

GFQ stands for Gait and Falls Questionnaire.

All participants were able to walk unassisted and had no history of dementia. Participants with PD had been diagnosed with idiopathic PD, and their disease duration was 10.1±5.5 years (ranging from 3 to 20 years). All participants provided their written informed consent. The experimental protocol (12–5462) was approved by the University Health Network Research Ethics Board (Toronto, Ontario, Canada) and carried out in accordance with the relevant guidelines and regulations.

### Motor and cognitive examination of participants with Parkinson’s Disease

All participants with PD were being treated with levodopa and were studied in the off-medication condition following overnight withdrawal from dopaminergic medications. We administered the motor section (Part III) of the UPDRS before the experimental task. After the experimental task, we administered the MoCA (Version 7.1).

On a separate day before the experiment, we administered the Gait and Falls Questionnaire [35] to four of the nine participants with PD that reported freezing of gait. The questionnaire quantified the severity of freezing and identified possible triggers of episodes.

### Experimental task

Each participant sat in a chair with a backrest and performed six runs of bilateral, cyclical ankle movements. The six runs alternated between being self-paced and externally paced by the sound of a metronome, with the first run always being externally paced (i.e., for each type of pacing, there were three runs). Each run lasted approximately one minute and was followed by a rest of approximately one minute.

Participants were instructed to maximally dorsiflex one foot and maximally plantarflex the other foot at each beat of the metronome (in an anti-phasic manner) without flexing or extending their toes. The metronome was set to 108 beats per minute (1.8 Hz), comparable to the cadence of normal overground walking [36]. For self-paced movements, the participants were instructed to replicate the rhythm of the metronome. The passive movements that resulted from the ankle movements (e.g., an upward movement of the knee as the foot dorsiflexed) were not constrained. Because the participants sat with their heels on an elevated footrest, the soles of their feet largely did not come into contact with any surface during the movement. Because the experimental task was performed with no resistance and supported heels, we assumed that the strength of contraction was relatively low with minimal effects of amplitude cancellation [37]. They were also instructed to focus their gaze on a bullseye, which was placed approximately 2 m in front of them in their line of sight as they sat upright and gazed forward. To minimize EEG artifacts, the participants were instructed to relax their upper body and to refrain from talking, swallowing, coughing, clenching their jaw, or blinking excessively. While the participants performed the movement, their EEG signals, EMG signals, and body kinematics were recorded.

### Data collection

All signals were recorded in epochs of approximately one minute, which began several cycles after the movement had been initiated and preceded the termination of the movement. The sampling of all signals was synchronized by an analogic switch, which sent a transistor-transistor logic signal that initiated the recording of kinematic data and EMG signals and timestamped the EEG signals.

To track the ankle movements, we used an optical motion capture system: a data acquisition device (MX Giganet, Vicon Motion Systems Ltd., United Kingdom), nine optical cameras (Bonita, Vicon Motion Systems Ltd., United Kingdom), and data acquisition software (Nexus 1.8.5, Vicon Motion Systems Ltd., United Kingdom). We placed 14-mm retroreflective markers over the EEG electrode locations, AF_7_ and AF_8_, and over the following bony landmarks: greater trochanter, lateral epicondyle of the femur, lateral malleolus and second metatarsal head on both sides. The instantaneous positions of the markers were recorded at 100 Hz.

EMG signals were recorded using a wireless EMG system (Trigno^™^ Wireless EMG System, Delsys Inc., United States). Each EMG sensor used 99.9%-silver electrodes, which were 1 mm in diameter and 5 mm in length. The electrodes were in bipolar configuration with inter-electrode spacing of 10 mm. The EMG sensors were placed bilaterally over the belly of the tibialis anterior muscle and the medial head of the gastrocnemius muscle. EMG signals were sampled at 2 kHz with a bandwidth of 20 Hz to 450 Hz and the common mode rejection ratio of over 80 dB.

EEG signals were recorded using an active electrode system (g.GAMMAsys, g.tec medical engineering GmbH, Austria) with compatible signal amplifiers (g.USBamp, g.tec medical engineering GmbH, Austria) and recording software (g.Recorder, g.tec medical engineering GmbH, Austria). According to the 10–10 system [38], we recorded from AF_z_, F_z_, F_1_, F_2_, F_3_, F_4_, FC_z_, FC_1_, FC_2_, FC_3_, FC_4_, C_z_, C_1_, C_2_, C_3_, C_4_, CP_z_, CP_1_, CP_2_, and Pz, which covered the midline primary sensorimotor cortex and its surrounding. EEG signals were sampled at 1.2 kHz using a monopolar montage with the reference electrode on the left ear lobe and the ground electrode over the right zygomatic process.

### Data analysis

Data analysis was performed offline using MATLAB R2016b (The MathWorks, Inc., United States). We quantified motor performance as the intra-individual mean and coefficient of variation of the following parameters: movement cycle duration, range of motion at the ankle, and the phase offset between the two feet. These parameters were selected to quantify the consistency and symmetry of the ankle movements. A movement cycle was defined such that dorsiflexion of the right foot was maximal at 0 and 100% of the cycle. The ankle angle was calculated between two lines: one line joining the markers over the lateral epicondyle of the femur and the lateral malleolus and another line joining the markers over the lateral malleolus and the second metatarsal head. The phase offset was calculated for angular velocities of the two ankles [16], such that the offset would be 180° for a symmetrically coordinated movement. For each participant, intra-individual mean and coefficient of variation of the above parameters were calculated across the minimum number of movement cycles that were completed among all participants after three epochs.

To assess the effects of head movements on EEG signals, we calculated the continuous wavelet transforms of the cyclical EEG signals at C_z_ using the complex Morlet wavelet. We also calculated the cyclical linear movements of the markers at the EEG electrode locations, AF_7_ and AF_8_ to assess the magnitude of head movements during the ankle movements.

For each epoch, EMG signals were centered by subtracting its mean and full-wave rectified to enhance the spectral power at the frequency of common input to the activated muscles [39,40]. To assess the effects of rectification, we estimated the power spectral densities of the cyclical EMG signals using Welch’s method. Cyclical EMG signals were down-sampled at 400 Hz and divided into eight sections of equal length with Hamming windows and 50% overlap. For each epoch, EEG signals were filtered by *i*) a second-order infinite impulse response notch filter, with a center frequency of 60 Hz and bandwidth of 1 Hz, and *ii*) a fourth-order Butterworth infinite impulse response filter, between 0.5 Hz and 100 Hz. For both processes, zero-phase digital filtering was used. The filtered EEG signals were decomposed by independent component analysis [41,42]. The resultant components and filtered EEG signals were examined for artifacts visually [43]. The contributions of components that contained artifactual waveforms were subtracted from the filtered EEG signals to generate noise-reduced EEG signals. The subtraction was restricted to the observed duration of artifactual waveforms to minimize the loss of information.

The noise-reduced EEG signals and rectified EMG signals were down-sampled at 400 Hz, and their coherence was calculated for each epoch using the complex Morlet wavelet:

$$\psi (t)=F_{b}\pi^{-0.5}e^{j2\pi F_{c}t}e^{-\frac{t^{2}}{F_{b}}},$$

where *j* is the imaginary unit, *F*_*b*_ is a bandwidth parameter, and *F*_*c*_ is the center frequency of the wavelet in Hz. The bandwidth parameter was set to 10, and the center frequency was set to 1.

Corticomuscular coherence was calculated as three-dimensional data across frequency and time. For each participant, the corticomuscular coherence (approximately one-minute long) was segmented into individual movement cycles, and the segments were used to calculate an ensemble average. Each ensemble average was calculated with the minimum number of cycles that were completed among all participants after three epochs.

The significance of each ensemble average was determined by a threshold value [10]:

$$SL=1-{\left[\frac{1}{N}\left(1-\frac{\alpha }{100}\right)\right]}^{\frac{1}{L-1}},$$

where *α* is the confidence level in percent, *L* is the number of segments that are used to calculate the ensemble average, and *N* is the number of data points (across frequency and time) in the ensemble average. The confidence level was set to 95%. Before applying the threshold, the ensemble average was binned across frequency and time, resulting in one pixel per Hz (between 1 and 100 Hz) and per percent of the movement cycle. The magnitude of corticomuscular coherence was calculated as the volume of significant coherence: the magnitude of coherence above the threshold at each pixel, integrated over the frequency-time plane of the ensemble average. A similar method has been used by Kilner et al. to quantify corticomuscular coherence [44]. For the volume of significant coherence, we also calculated its center frequency as the geometric centroid along frequency.

The cyclical patterns of corticomuscular coherence at C_z_ were validated using surrogate coherence. For each participant, an ensemble average of coherence was calculated by pairing the *i*^th^-cycle segment of the EEG signal with the *j*^th^-cycle segment of an EMG signal, such that *i* ≠ *j*. For each participant, such ensemble averages were calculated 100 times with differently permutated pairing of EEG and EMG signals, and the average magnitude of the 100 ensemble averages was used as surrogate coherence. Patterns of coherence, which were present in experimental coherence but abolished in surrogate coherence, were considered valid as these patterns indicate the synchronization between EEG and EMG signals that does not relate to the mere power of the signals. We chose this approach to eliminate only the cyclical pairing between the EEG and EMG signals. The validation was only performed at C_z_ because it was the most relevant electrode location (i.e., over the midline primary sensorimotor cortex).

For the intra-individual mean and coefficient of variation of cycle duration and range of motion, we performed 3-way ANOVA with the *i*) presence of PD (present or absent), *ii*) type of pacing (self- or external pacing), and *iii*) side of body (left or right) as factors. For the intra-individual mean and coefficient of variation of the phase offset, we performed 2-way mixed-design ANOVA with the *i*) presence of PD as a between-subject factor and *ii*) type of pacing as a within-subject factor. To eliminate redundancy, we only analyzed the phase offset that was calculated with the right ankle as the leading side.

On the volume and center frequency of significant coherence at C_z_, we performed 4-way ANOVA with the *i*) presence of PD, *ii*) type of pacing, *iii*) side of body, and *iv*) muscle (tibialis anterior or medial gastrocnemius muscles) as factors. To examine the cortical distribution of coherence, we performed 5-way ANOVA on the volume of significant coherence with the *i*) presence of PD, *ii*) type of pacing, *iii*) side of body, *iv*) muscle, and *v*) EEG electrode location as factors.

We examined how surrogate and experimental coherence at C_z_ differed in magnitude by performing 2-way ANOVA with *i*) the presence of PD and *ii*) type of coherence (experimental or surrogate) as factors. Preliminarily, we had observed that surrogate coherence showed relatively high magnitudes of coherence below 6 Hz. Thus, the 2-way ANOVA was performed separately above and below 6 Hz.

For significant main effects, we performed *post hoc* multiple comparison tests with Tukey’s honestly significant difference criterion. The significance level was set to 5% for all tests. We also performed multiple-sample tests for equal variances (Bartlett’s test) and Royston’s multivariate normality tests [45] on the data for ANOVA.

## Results

### Motor and cognitive examination of participants with Parkinson’s Disease

For participants with PD, 11.9±1.7 hours had elapsed since their last dose. The motor scores of the Unified Parkinson’s Disease Rating Scale (UPDRS) were 17.6±6.6 (out of 108), with a higher value indicating greater motor impairment. The leg agility subscores were 1.2±0.9 (out of 4) for the right and 1.3±0.9 for the left.

The Montreal Cognitive Assessment (MoCA) scores were 26.9±1.83 (out of 30), with a lower value indicating greater cognitive impairment. The scores for the Freezing of Gait Questionnaire, which is a subset of the Gait and Falls Questionnaire [35], were 9.0±1.6 (out of 24), with a higher score indicating greater severity of freezing. Of the four participants with PD that reported freezing of gait, three reported start hesitation and one reported freezing while walking straight.

### Motor performance of ankle movements

For each type of pacing, healthy participants completed 167±8 cycles and participants with PD completed 166±21 cycles after three one-minute runs. Among all participants, the minimum number of completed cycles was 111. The inter-run rest was 80.6±30.7 seconds for healthy participants and 116±23 seconds for participants with PD.

**Fig 1:**
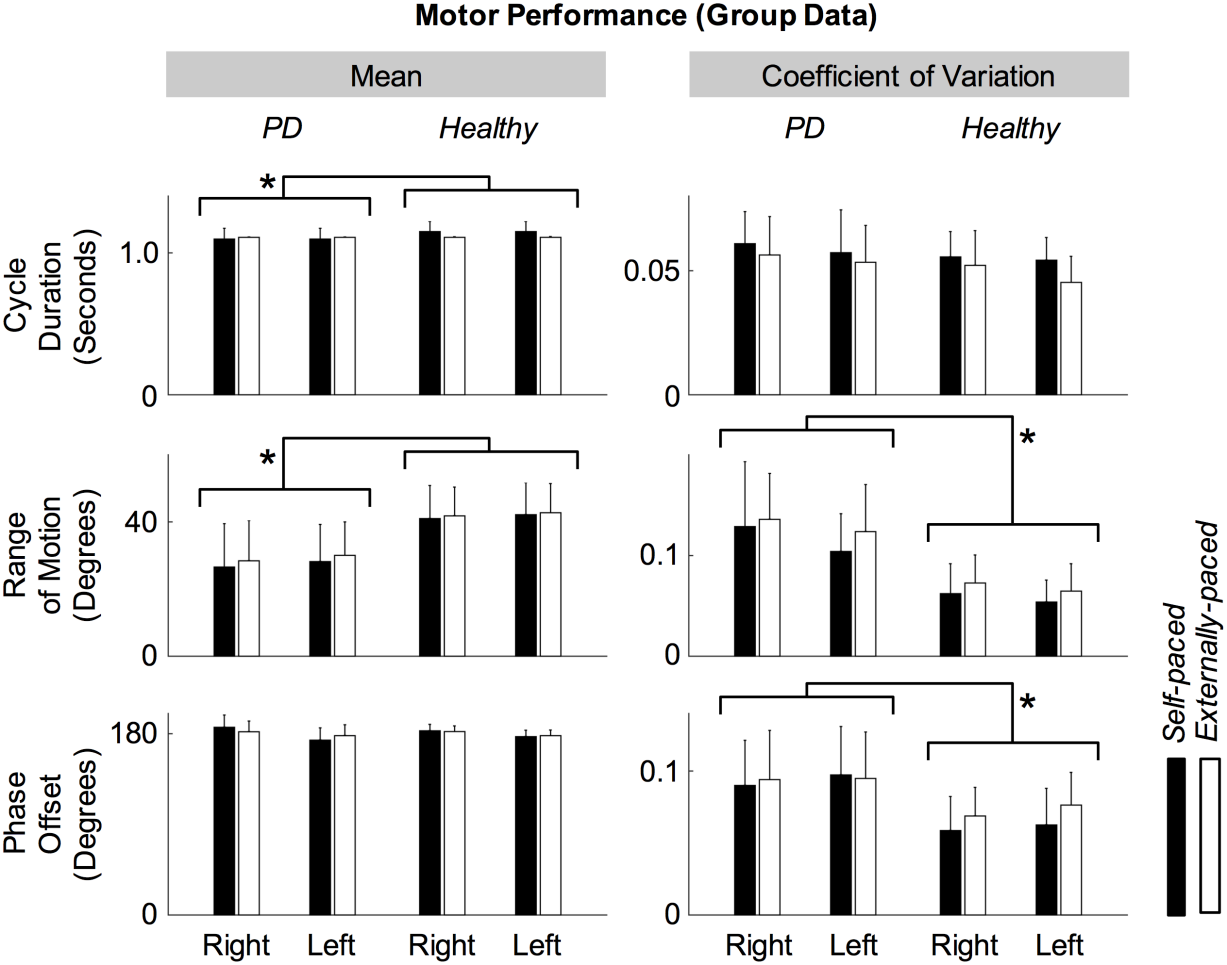
Intra-individual mean and coefficient of variation of motor performance parameters for self-paced and externally-paced movements. Error bars indicate inter-individual standard deviations.

Fig 1 compares the motor performance of the two groups during the ankle movements. The intra-individual mean of cycle duration was significantly shorter for participants with PD (*F*_1,65_ = 4.89, *p* = .0305) but was not significantly affected by the type of pacing (*F*_1,65_ = 1.35, *p* = .250) or side of body (*F*_1,65_ < 0.001, *p* = .994). The coefficient of variation of cycle duration was not significantly affected by the presence of PD (*F*_1,65_ = 2.73, *p* = .103), type of pacing (*F*_1,65_ = 2.78, *p* = .100), or side of body (*F*_1,65_ = 1.42, *p* = .237). The intra-individual mean of the range of motion was significantly smaller for participants with PD (*F*_1,65_ = 31.4, *p* < .001) but was not significantly affected by the type of pacing (*F*_1,65_ = 0.270, *p* = .605) or side of body (*F*_1,65_ = 0.295, *p* = .589). The coefficient of variation of the range of motion was significantly more variable for participants with PD (*F*_1,65_ = 41.4, *p* < .001) but was not significantly affected by the type of pacing (*F*_1,65_ = 1.68, *p* = .199) or side of body (*F*_1,65_ = 2.03, *p* = .159). The intra-individual mean of the phase offset was not significantly affected by the presence of PD (*F*_1,32_ = 0.295, *p* = .591) or type of pacing (*F*_1,32_ = 0.784, *p* = .383). The coefficient of variation of the phase offset was significantly more variable for participants with PD (*F*_1,32_ = 7.67, *p* = .00928) but not significantly affected by the type of pacing (*F*_1,32_ = 0.340, *p* = .564). According to multiple-sample tests for equal variances and Royston’s multivariate normality tests, the mean cycle duration was neither homogeneous (*T* = 149.37, *p* < .001) nor normal (*H* = 34.53, *p* < .001), the coefficient of variation of cycle duration was homogeneous (*T* = 5.28, *p* = .626) and normal (*H* = 5.15, *p* = .639), the mean range of motion was homogeneous (*T* = 2.36, *p* = .937) and normal (*H* = 3.83, *p* = .320), the coefficient of variation of the range of motion was not homogeneous (*T* = 14.20, *p* = .048) but normal (*H* = 3.78, *p* = .786), the mean phase offset was homogeneous (*T* = 6.03, *p* = .110) and normal (*H* = 2.85, *p* = .524), and the coefficient of variation of the phase offset was homogeneous (*T* = 1.59, *p* = .663) and normal (*H* = 2.14, *p* = .694).

Among the factors of analysis of variance (ANOVA) on the parameters of motor performance, the only significant interaction was between the presence of PD and type of pacing on the intra-individual mean of cycle duration (*F*_1,65_ = 4.59, *p* = .0360), indicating that cycle durations of healthy participants were more affected by the type of pacing.

For both participant groups, regardless of the type of pacing, markers at AF_7_ and AF_8_ were within a volume of approximately 1 cm^3^ during each movement cycle. For healthy participants, the average linear head movement was less than 8 mm, 7 mm, and 4 mm, in the anteroposterior, mediolateral, and longitudinal directions, respectively. The equivalent measures for participants with PD were less than 5 mm, 4 mm, and 2 mm.

### Cyclical corticomuscular coherence during ankle movements

**Fig 2:**
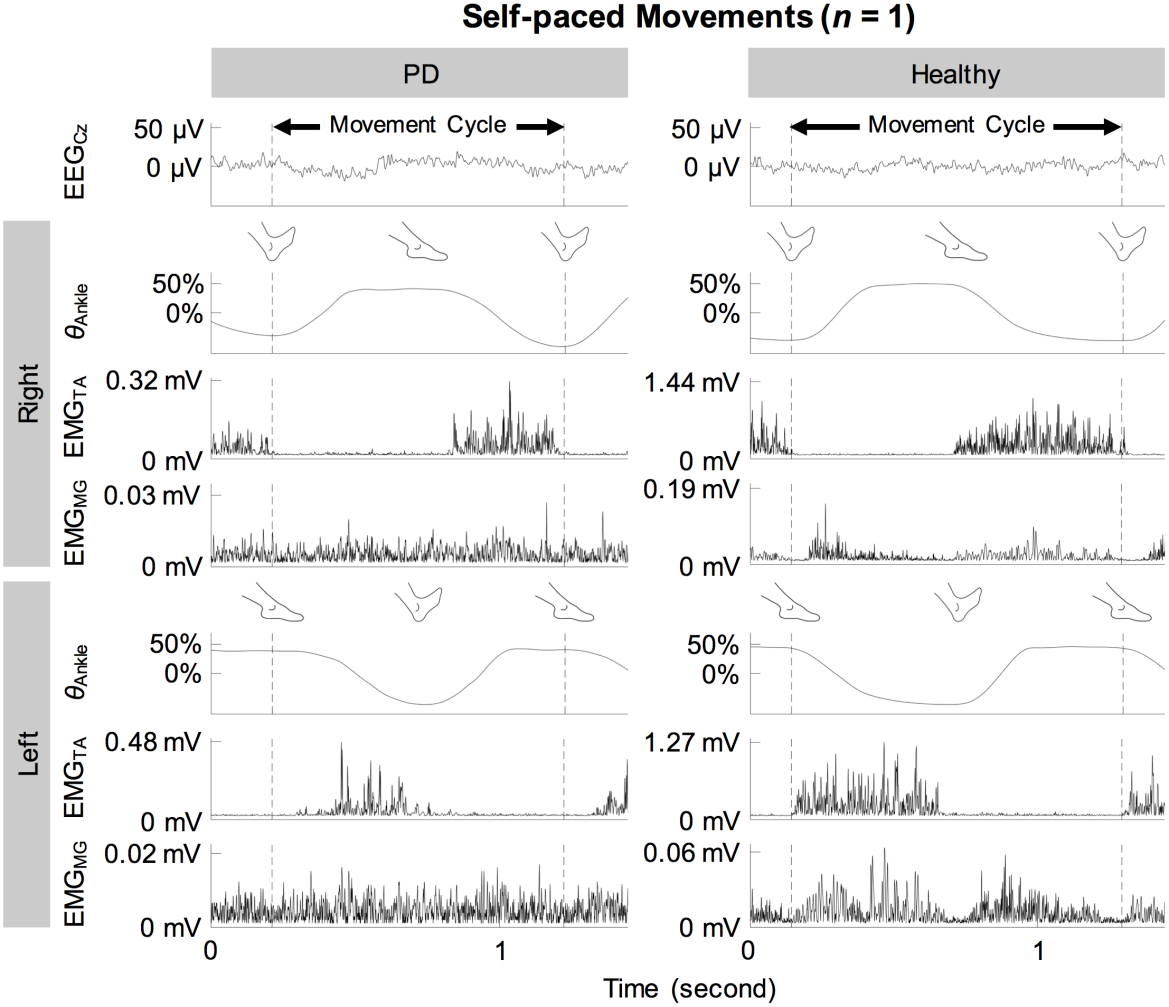
EEG, kinematic, and EMG signals of representative participants during self-paced ankle movements. Noise-reduced EEG signal from C_z_ (EEG_Cz_), ankle angle (*θ*_Ankle_), and full-wave rectified EMG signals from the tibialis anterior (EMG_TA_) and medial gastrocnemius (EMG_MG_) muscles are shown. Ankle angles have been centered and normalized to its range.

**Fig 3:**
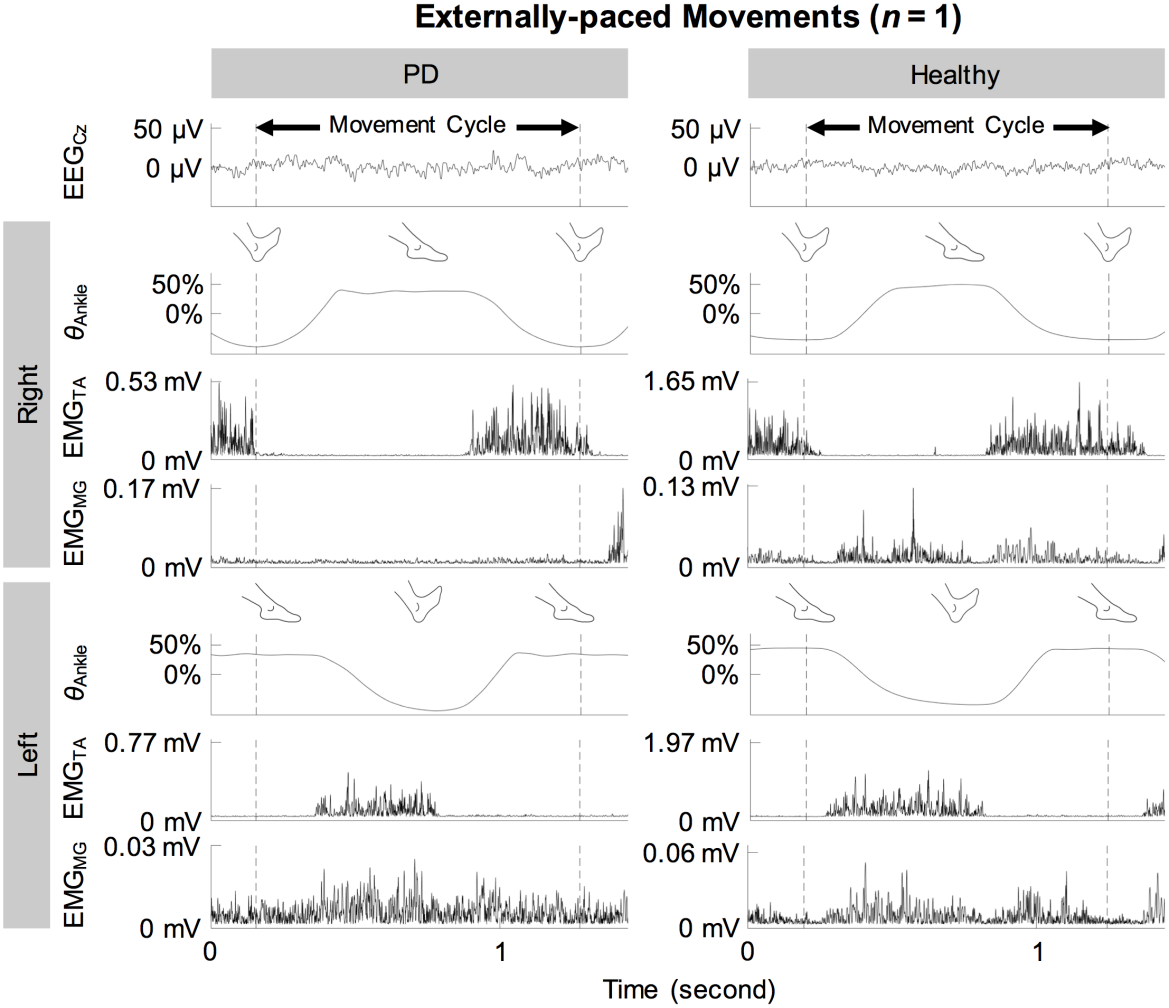
EEG, kinematic, and EMG signals of representative participants during externally-paced ankle movements. Noise-reduced EEG signal from C_z_ (EEG_Cz_), ankle angle (*θ*_Ankle_), and full-wave rectified EMG signals from the tibialis anterior (EMG_TA_) and medial gastrocnemius (EMG_MG_) muscles are shown. Ankle angles have been centered and normalized to its range.

**Fig 4:**
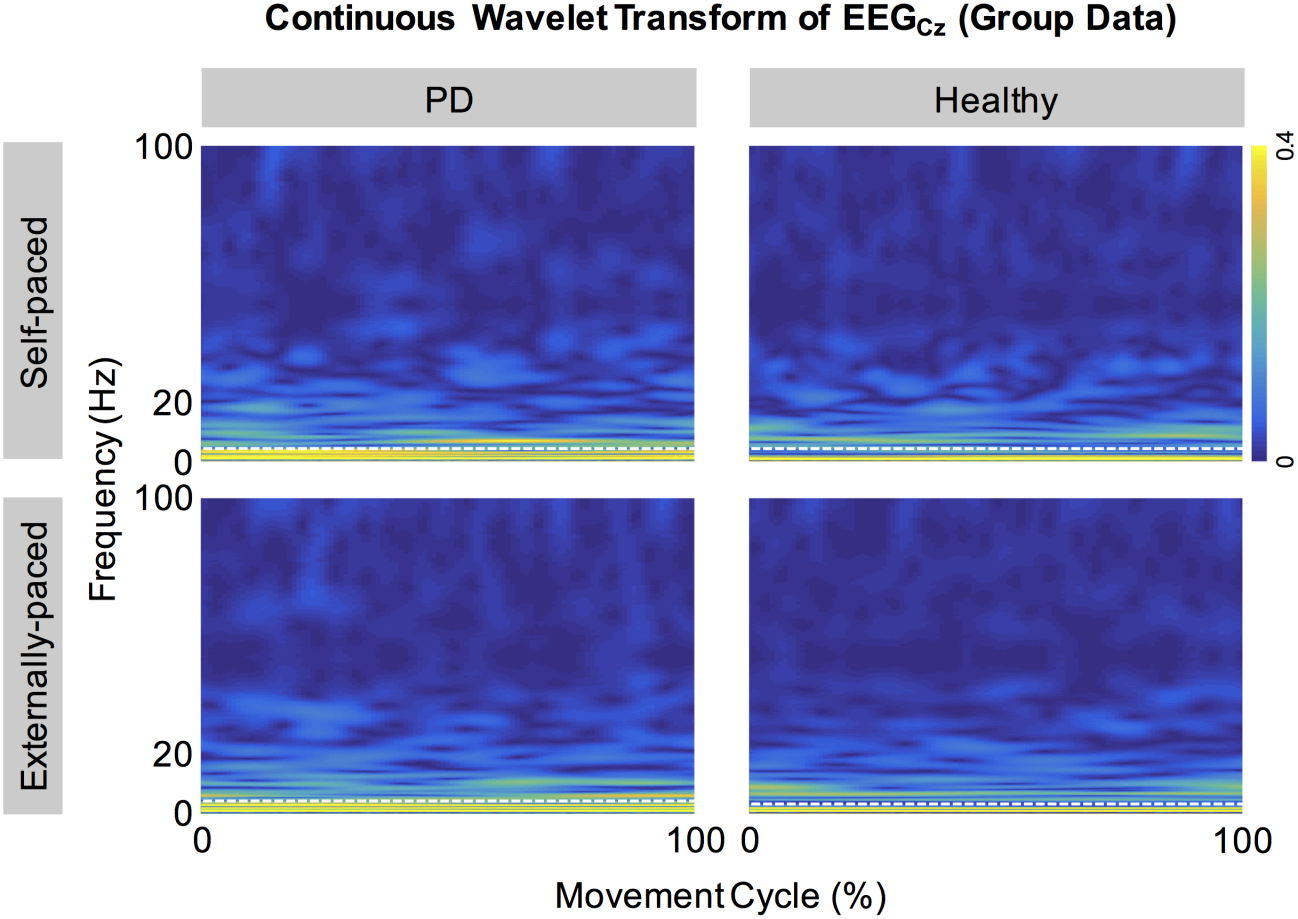
Continuous wavelet transforms of cyclical EEG signals at C_z_. Group averages are shown. The white dotted lines indicate 5 Hz.

**Fig 5:**
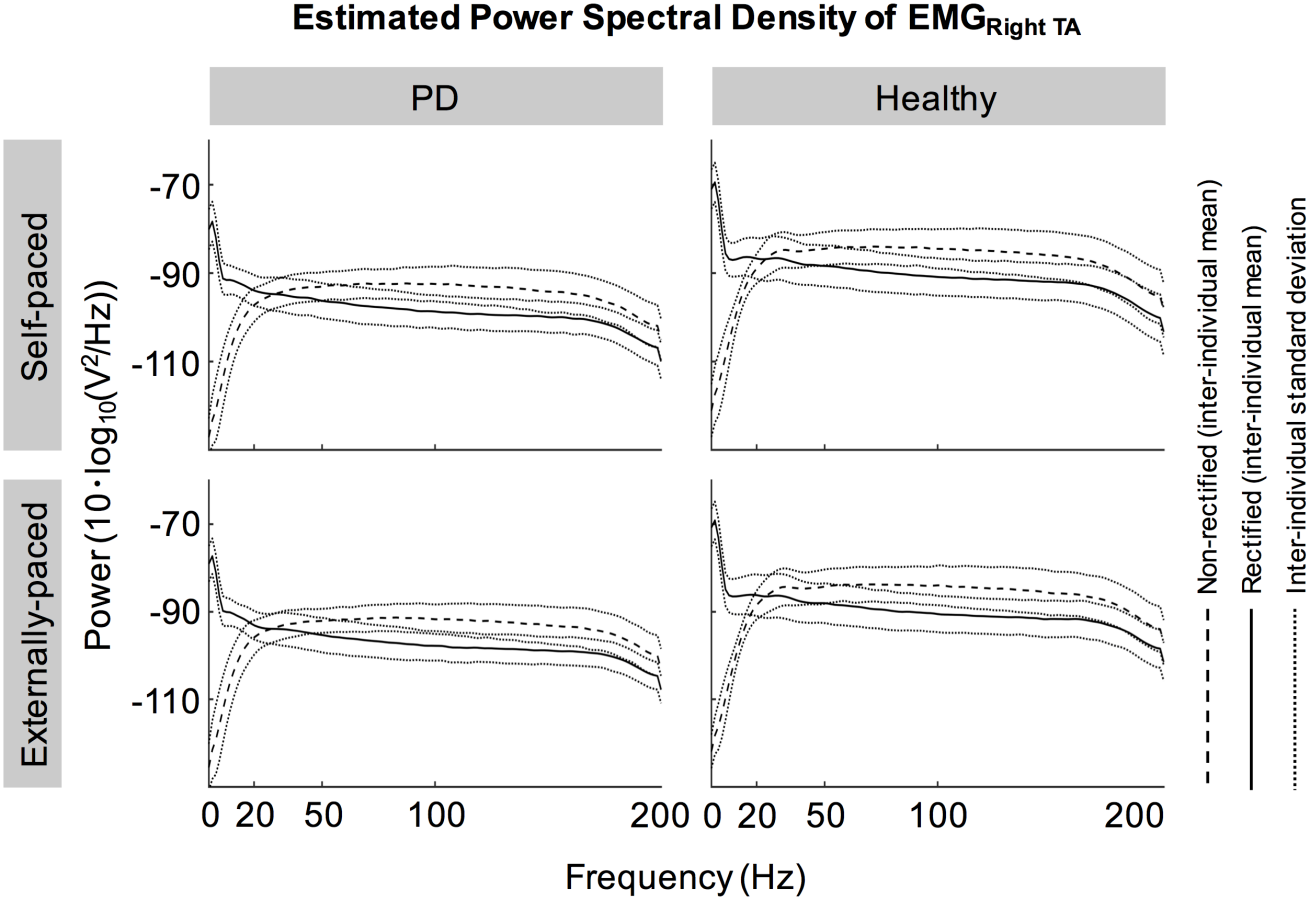
Estimated power spectral densities of EMG signals from the right tibialis anterior (TA) muscle.

**Fig 6:**
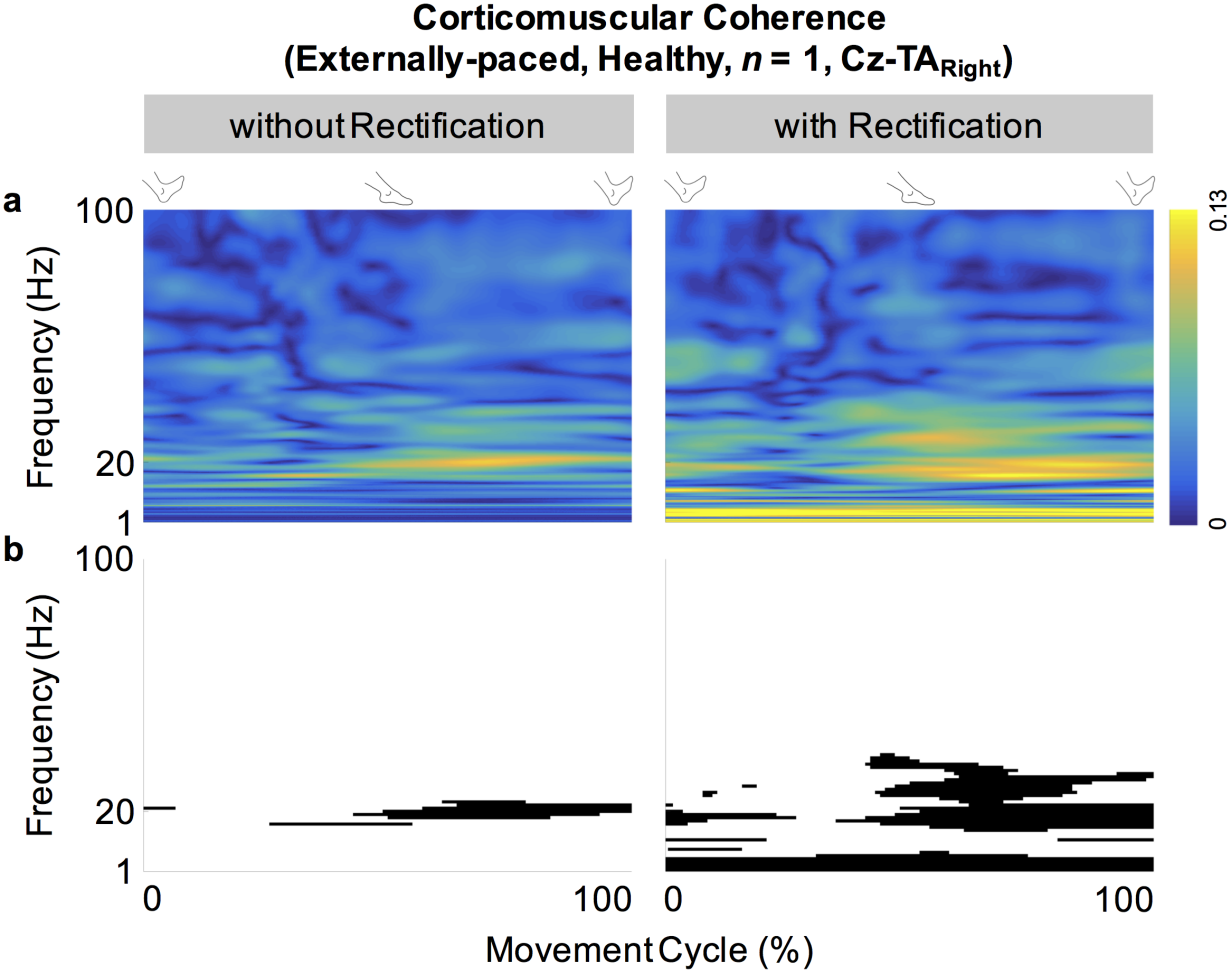
Effects of rectifying EMG signals on cyclical corticomuscular coherence. (a) Cyclical coherence between C_z_ and the right tibialis anterior (TA) muscle of a healthy participant during externally-paced movements. (b) Significant portions of the cyclical coherence in (a).

Fig 2 shows the full-wave rectified EMG signals from representative participants during self-paced movements. Both participants showed cyclical increase in the activation of the tibialis anterior muscles during dorsiflexion and relatively weak activation of the medial gastrocnemius muscles. These observations were also true for externally-paced movements (Fig 3). The continuous wavelet transforms of EEG signals at C_z_ did not show observable peaks at harmonics of the movement frequency (1.8 Hz) above 5 Hz (Fig 4). Fig 5 shows how the full-wave rectification modulated the estimated power spectral densities of EMG signals, and Fig 6 shows the corresponding change in the pattern of corticomuscular coherence around 20 Hz.

**Fig 7:**
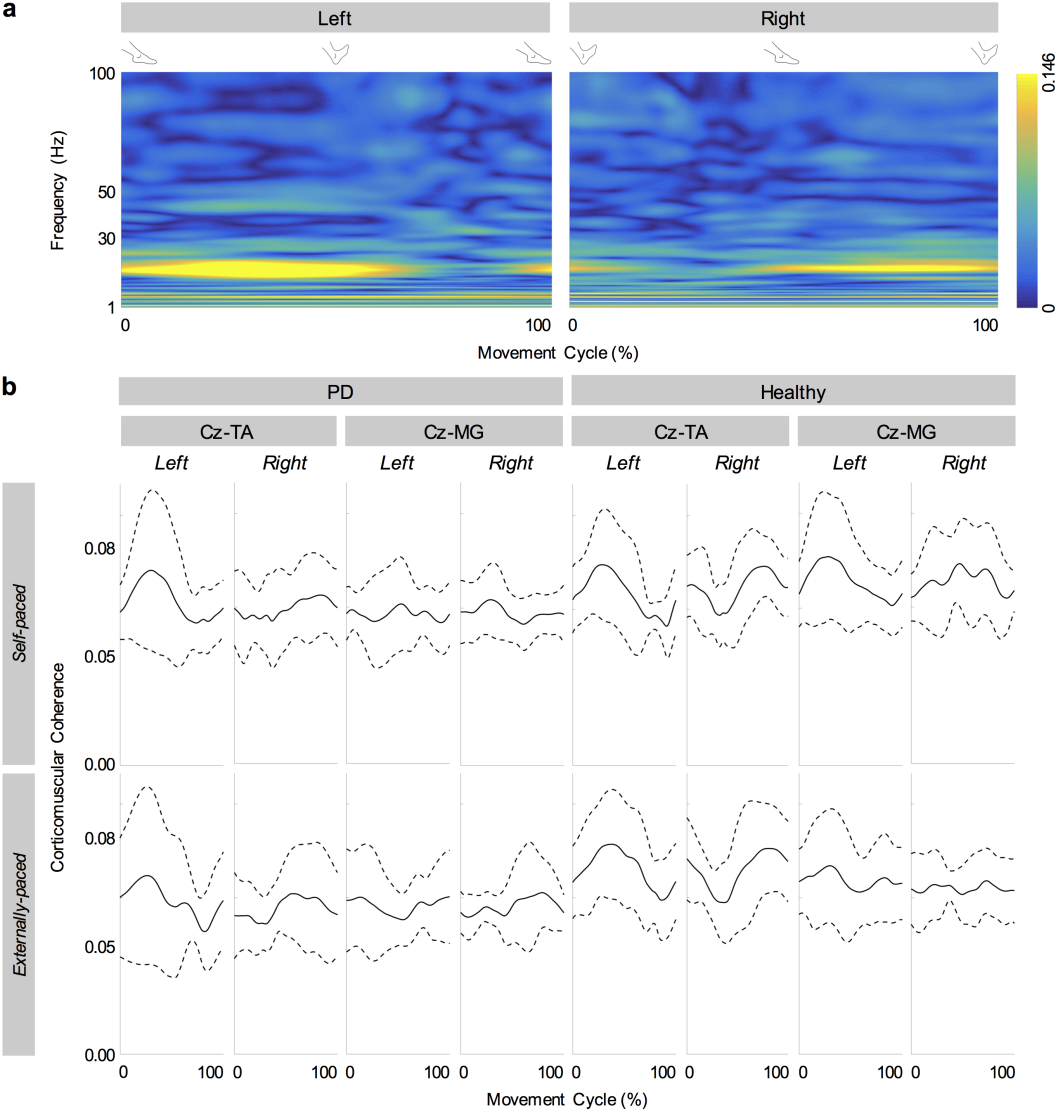
Cyclical corticomuscular coherence. (a) Cyclical coherence between C_z_ and tibialis anterior muscles of a participant with PD during externally-paced movements. (b) Group averages of cyclical corticomuscular coherence in the beta band (13 to 30 Hz). Coherence is calculated between C_z_ and the tibialis anterior (TA) and medial gastrocnemius (MG) muscles. Solid lines indicate inter-individual mean and dotted lines indicate inter-individual standard deviations.

Fig 7a shows the cyclical coherence between C_z_ and the tibialis anterior muscles of a participant with PD during externally-paced movements. Within the movement cycle, corticomuscular coherence increased dynamically in the beta band, coinciding with ankle dorsiflexion (*cf*. Fig 2). Fig 7b shows the group average of cyclical corticomuscular coherence in the beta band. Generally, between C_z_ and the tibialis anterior muscles, corticomuscular coherence in the beta band increased cyclically during dorsiflexion (*cf*. Fig 2). Volumes of significant corticomuscular coherence were centered about the beta band (Table 2). The cyclical patterns of coherence were less consistent between C_z_ and the medial gastrocnemius muscles (Fig 7b).

**Table 2:** Volume and center frequency of significant coherence between EEG signal from C_z_ and EMG signals from the tibialis anterior (TA) and medial gastrocnemius (MG) muscles.

Group	Measurement	Muscle	Self-pacing		External Pacing	
			Left	Right	Left	Right
Healthy	Volume (Hz·%_*Movement Cycle*_)	TA	6.44±7.60	4.53±5.91	6.93±5.17	8.37±9.87
		MG	5.11±4.23	5.23±3.77	3.91±3.58	3.44±2.46
	Center Frequency (Hz)	TA	15.4±4.7	17.2±4.7	16.7±4.9	17.2±6.9
		MG	14.8±3.5	14.9±4.5	14.2±4.6	14.8±6.1
PD	Volume (Hz·%_*Movement Cycle*_)	TA	6.78±7.47	4.61±4.03	6.19±9.69	4.20±3.30
		MG	4.20±3.65	2.41±1.70	3.79±5.07	3.39±2.65
	Center Frequency (Hz)	TA	15.0±5.8	14.7±6.0	14.6±6.4	18.4±9.6
		MG	12.4±4.0	16.7±4.3	16.2±4.8	16.4±5.7

Each entry shows the inter-individual mean±standard deviation. The values did not significantly differ between groups.

Table 2 summarizes the volume and center frequency of significant corticomuscular coherence for the two groups. At C_z_, the volume of significant coherence was significantly affected by the muscle (*F*_1,133_ = 5.12, *p* = .0253) but not by the presence of PD (*F*_1,133_ = 1.31, *p* = .254), type of pacing (*F*_1,133_ = 0.0160, p = .900), or side of body (*F*_1,133_ = 0.954, *p* = .330). *Post hoc* analysis revealed that the volume of significant coherence between C_z_ and the tibialis anterior muscles was larger than that between C_z_ and the medial gastrocnemius muscles. The center frequency was not significantly affected by the presence of PD (*F*_1,133_ = 0.0163, *p* = .899), type of pacing (*F*_1,133_ = 0.978, *p* = .325), muscle (*F*_1,133_ = 1.37, *p* = .244), or side of body (*F*_1,133_ = 2.17, *p* = .143). Neither for the volume nor the center frequency of significant coherence did the factors of ANOVA interact significantly. According to multiple-sample tests for equal variances and Royston’s multivariate normality tests, the magnitude of coherence was neither homogeneous (*T* = 53.04, *p* < .001) nor normal (*H* = 61.68, *p* < .001). The same tests indicated that the frequency of coherence was homogeneous (*T* = 14.35, *p* = .499) but not normal (*H* = 21.53, *p* = .029).

**Fig 8:**
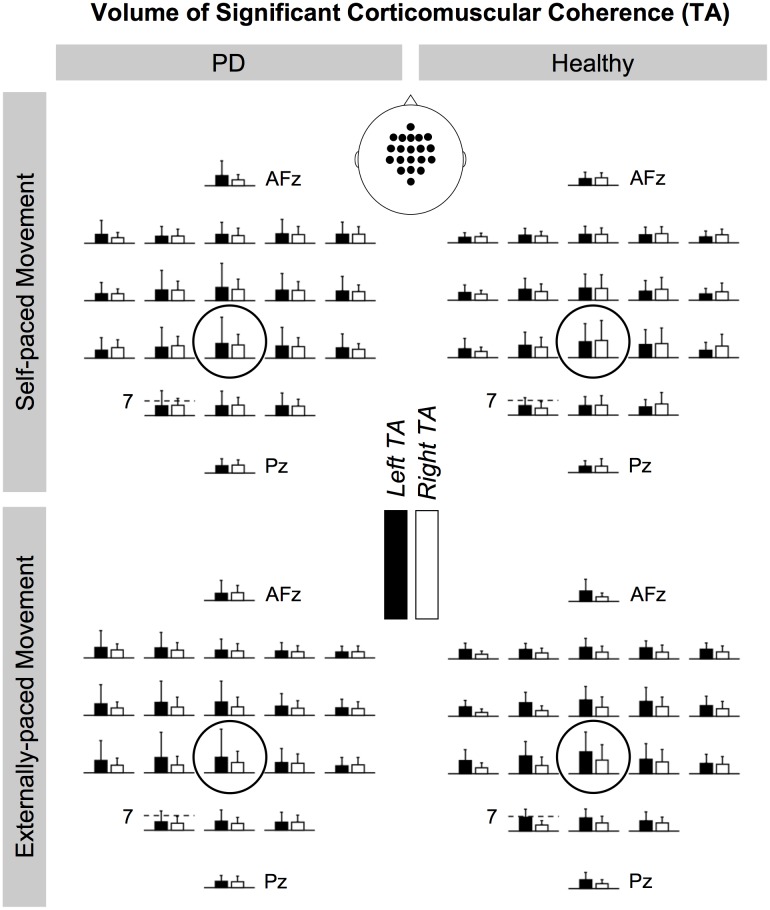
Cortical distributions of significant beta-band corticomuscular coherence for the tibialis anterior (TA) muscles. All bars show the volume of corticomuscular coherence in the units of Hz**·**%_*Movement Cycle*_. C_z_ is circled. The scale of the vertical axis is the same across electrode locations, and the magnitude of the bar graphs is indicated at CP_1_. The rostral direction is towards the top of the page.

**Fig 9:**
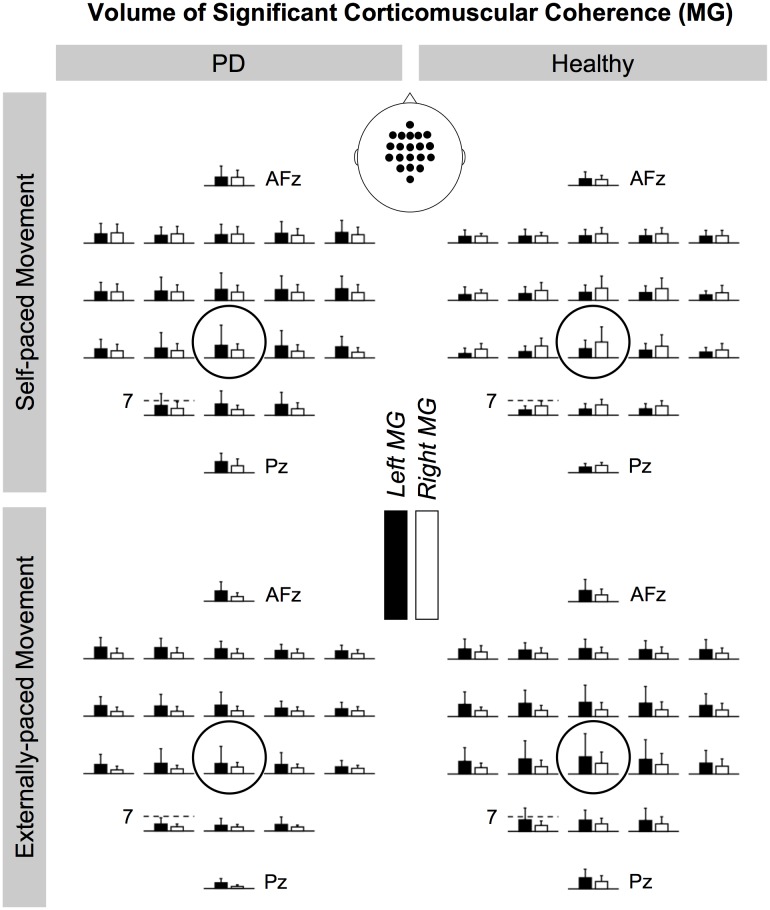
Cortical distributions of significant beta-band corticomuscular coherence for the medial gastrocnemius (MG) muscles. All bars show the volume of corticomuscular coherence in the units of Hz**·**%_*Movement Cycle*_. C_z_ is circled. The scale of the vertical axis is the same across electrode locations, and the magnitude of the bar graphs is indicated at CP_1_. The rostral direction is towards the top of the page.

Fig 8 shows the cortical distributions of the volume of significant coherence in the beta band between EEG signals and EMG signals from the tibialis anterior muscles. Fig 9 shows the same distributions for the medial gastrocnemius muscles. Generally, the cortical distribution peaked at C_z_ although this pattern appeared to be more distinct for the tibialis anterior muscles. ANOVA shows that the magnitude of coherence at C_z_ was significantly larger than the magnitude at all other locations in 89% of the conditions (i.e., combinations between the factors of ANOVA). This was followed by the magnitude at FC_z_, which was larger than the magnitude at all other locations in 54% of the conditions, and the magnitude at C_1_, which was larger than the magnitude at locations other than C_z_ and C_2_ in 44% of the conditions. The analysis did not show PD-related differences in the cortical distribution of coherence.

### Validation of experimental corticomuscular coherence

**Fig 10:**
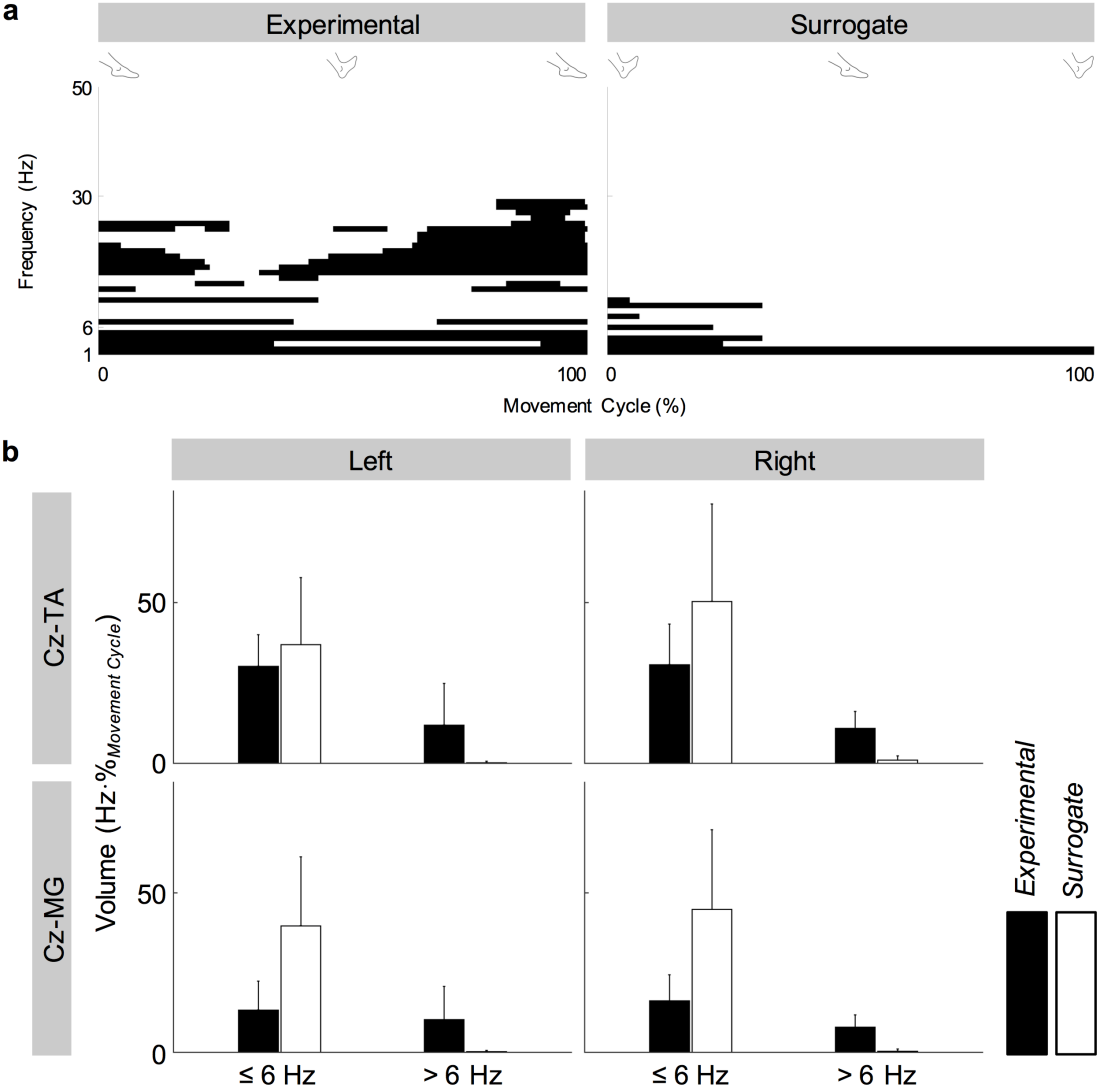
Validation of experimental corticomuscular coherence. (a) Significant experimental and surrogate coherence between C_z_ and the right tibialis anterior muscle of a participant with PD during externally-paced movements. Pixels with significant coherence are shown in black. (b) Volumes of significant coherence between C_z_ and the tibialis anterior (TA) and medial gastrocnemius (MG) muscles of participants with PD during self-paced movements. Error bars indicate inter-individual standard deviations.

Fig 10 illustrates the validation of experimental coherence using surrogate coherence. Patterns of significant experimental coherence were preserved in surrogate coherence below 6 Hz but were abolished above 6 Hz. Below 6 Hz, the volume of significant coherence was significantly affected by the presence of PD (*F*_1,284_ = 31.2, *p* < .001) and surrogation of coherence (*F*_1,284_ = 45.5, *p* < .001). *Post hoc* analysis revealed that the volume of significant coherence was larger for healthy participants and for surrogate coherence. Above 6 Hz, the volume of significant coherence was significantly affected by the surrogation of coherence (*F*_1,284_ = 171, *p* < .001) but not by the presence of PD (*F*_1,284_ = 1.445, *p* = .230). *Post hoc* analysis revealed that the volume of significant coherence was smaller for surrogate coherence.

Below 6 Hz, the presence of PD and type of coherence interacted significantly (*F*_1,284_ = 4.61, *p* = .0326), indicating that the difference in the volume of significant coherence between experimental and surrogate coherence was larger for participants with PD. Above 6 Hz, the interaction between the two factors was not significant (*F*_1,284_ = 1.69, *p* = .194).

## Discussion

Participants with PD exhibited several kinematic abnormalities during the ankle movements: faster movement frequencies (especially with self-pacing), reduced and more variable range of motion, and more variable bilateral coordination compared to healthy participants (Fig 1). Similar increase in movement frequency has been reported for self-paced finger tapping at a specified frequency [46–50], reduced range of motion has been reported for walking [36] and self-paced finger tapping [51,52], and increased variability has been reported for various parameters of gait in PD [1–4]. Tests for homogeneity and normality indicated that the observed abnormalities in participants with PD were valid for the reduced range of motion and more variable bilateral coordination. Thus, the consistency and symmetry of the ankle movements differed significantly between participants with and without PD.

The observed kinematic abnormalities may have been caused partially by the requirement for anti-phasic coordination. Compared to healthy individuals, anti-phasic movements in PD can transition spontaneously into in-phasic movements at lower frequencies [15], exhibit greater asymmetry [14,16,17,53], induce freezing [53], or simply fail [18,19]. Although our participants with PD did not exhibit these abnormalities, the existing evidence suggests that individuals with PD experience difficulty with performing anti-phasic movements.

In addition to anti-phasic coordination, participants with PD may have had deficits in learning and automatizing the ankle movements. In monkeys and humans, the basal ganglia appears to participate in learning an unfamiliar motor task [54–57] and automatizing its execution [55,58–61]. Although the ankle movements were relatively simple, impaired motor learning and task automatization could have contributed to the observed kinematic abnormalities.

In participants with and without PD, coherence between C_z_ and the tibialis anterior muscles increased cyclically in the beta band during dorsiflexion of the feet (Fig 7b). This increase occurred bilaterally and regardless of the type of pacing (Fig 7b). Furthermore, these patterns of coherence were validated using surrogate coherence. With the shuffled pairing between cycles of EEG and EMG signals, the volume of significant coherence above 6 Hz significantly decreased and became almost negligible (Fig 10). Thus, the patterns of coherence above 6 Hz could be attributed to the cyclical ankle movements. Conversely, the volume of significant coherence below 6 Hz significantly increased with the shuffled pairing (Fig 10). Thus, coherence below 6 Hz was not validated. The patterns of coherence were less distinct between C_z_ and the gastrocnemius muscles (Fig 7b), possibly due to the relative absence of phasic activation of the gastrocnemius muscles (Fig 2). We observed that full-wave rectification of EMG signals enhanced their power (Fig 5) and the pattern of corticomuscular coherence (Fig 6) around 20 Hz. Similar modulation of the power spectrum by full-wave rectification has been reported previously [62]. Although this frequency was at the lower threshold of the bandwidth of the EMG system, rectification appears to amplify the power at the common motor unit recruitment frequency based on a broad spectrum of the original, unrectified signal. Such behavior has been supported by experimental evidence [40] and computational modeling [37,39].

For the tibialis anterior muscles, the cortical distributions of significant beta corticomuscular coherence generally peaked at C_z_ (Fig 8). Such somatotopy has been observed for the tibialis anterior muscle during isometric contractions [63]. The cortical distributions were less distinct for the medial gastrocnemius muscles (Fig 9). Again, this may have been due to the muscle being less active than the tibialis anterior muscle.

The minimal influence from movement artifacts was suggested by the absence of peaks in the estimated power spectral densities of cyclical EEG signals at C_z_ (Fig 4): during tasks that induce substantial electrode movements, artifacts can be present in EEG signals at the movement frequency and its harmonics [64]. The absence of significant artifacts was also indirectly supported by the kinematic data, as the head markers stayed within a space of approximately 1 cm^3^ during each movement cycle.

Contrary to our hypothesis, the magnitude of corticomuscular coherence did not significantly differ between the two groups (Table 2). As participants with PD exhibited several kinematic abnormalities (Fig 1), the lack of group discrepancy in the magnitude of coherence suggests that the pathological processes, which impaired motor performance, occurred outside linear corticomuscular communication or that changes in neural correlates maintained corticomuscular communication but not motor performance.

Whichever the case, it is likely that pathological processes that affected motor performance involved the basal ganglia, which is implicated in many aspects of motor control [27] and is affected by neuronal degeneration in PD [24–26]. Pathological activities within the ganglia may affect motor control via the recipients of the basal ganglia output: the ventral anterior and ventrolateral nuclei of the thalamus, which project back to the motor cortex [27,65,66], or the pedunculopontine nucleus (PPN). Abnormal cortico-basal ganglia interaction has been observed in rat models of PD [31,32] and individuals with PD [33,34] although its exact implications for the ankle movements are unknown. As for the basal ganglia output to the PPN, this may particularly affect the performance of anti-phasic ankle movements. The PPN comprises the mesencephalic locomotor region [67–69], which is implicated in initiating and sustaining locomotive actions [70–72]. The notion that PD involves pathological oscillations within the PPN, which contribute to the impairment of locomotive actions, is supported by the finding that deep brain stimulation of the PPN improves gait in individuals with PD in the off-medication condition [73,74]. Also, in individuals with PD, brisk ankle movements modulate oscillations in the PPN and cortical-PPN coherence in the beta band [75]. Although the ankle movements in this study were substantially different from locomotion, they share some key functional requirements with locomotion such as the maintenance of rhythm and anti-phasic coordination of the lower limbs. Thus, pathological output from the basal ganglia may have affected the performance of the ankle movements via the PPN and a subsequent pattern generating neuronal circuit at the spinal level [76,77].

Several studies indicated that the neural correlates of cyclical hand movements are affected by PD [13,47,78]. Particularly, during an anti-phasic bimanual task, individuals with PD show greater activation of the primary motor cortex and less activation within the basal ganglia than healthy individuals [13]. Although our experimental task involved lower limbs, the functional requirement (of performing a bilateral, anti-phasic movement) was similar to that of the aforementioned study, as was the disease severity of the participants [13]. Thus, it is possible that our participants with PD also recruited neural correlates that differed from those of healthy participants. However, the exact changes in neural correlates could not be determined without additional studies. Identifying the neural correlates in individuals with and without PD may help delineate how linear corticomuscular communication is maintained during the ankle movements.

Our observations differed from the results of a previous study, which found decreased beta coherence in PD during sustained isometric wrist extension [20]. Such discrepancy may have been due to the difference in the tasks: sustained isometric contraction compared to cyclical movements. Compared to cyclical movements, sustained contractions may require greater conscious control of the level of muscle activation, thus inducing greater participation by the primary sensorimotor cortex. It has also been shown that, between isometric and dynamic concentric plantarflexion with comparable ankle angles and forces, the motor unit discharge rate is significantly higher during dynamic plantarflexion [79], suggesting that the nature of contraction affects how motor units are recruited. If isometric and dynamic contractions differ substantially in how they are controlled, then it is possible that corresponding corticomuscular communication is differentially vulnerable to PD-related changes during isometric and dynamic contractions. The discrepancy between our findings and the previous study [20] may have also been related to the difference between upper and lower limb muscles, with upper limb muscle receiving stronger corticospinal projections [80]. Because of the stronger projections, upper limb muscles may rely more on corticospinal communication during contractions. Assuming that such communication is reflected in corticomuscular coherence, tasks with greater corticospinal communication may be more affected by PD.

Corticomuscular coherence can be affected in diseases other than PD. It has been reported that stroke can significantly decreases the magnitude of beta corticomuscular coherence on the affected side [81] and shift the location of maximum beta corticomuscular coherence away from the expected location: contralateral sensorimotor cortex [82]. Cerebral palsy has been associated with increased magnitude of beta corticomuscular coherence [83]. We did not observe such phenomena in PD. Although our findings differed from those of previous studies, several discrepancies make the comparison difficult. The main discrepancies are in the experimental task and pathophysiology. The studies on stroke used sustained wrist extension or a gripping task with visual feedback of force production [81,82], and the study on cerebral palsy used externally-cued ballistic hand movements [83]. In the stroke studies, only a small percentage of participants (2 of 6 participants or 3 of 25 participants) showed lesions in the basal ganglia [81,82]. In the cerebral palsy study, it is uncertain how much the interaction between the basal ganglia and the sensorimotor cortex is affected.

Despite reported evidence that aural pacing evokes synchronized periodic fields in the primary auditory cortex [84] and that increased attention or effort increases corticomuscular coherence [44,85–92], we did not observe any significant effects of aural pacing on the magnitude of coherence.

This study had several limitations. As we used coherence, our analysis focused on the linear aspect of corticomuscular communication. Because of the complex interconnections that the primary motor cortex forms with adjacent cortical areas and subcortical structures [93], the linear aspect alone probably cannot comprehensively capture how PD affects corticomuscular communication. Indeed, non-linear communication is likely if pathological signals are transmitted from the basal ganglia to the spinal cord as speculated above. Recently, Yang et al. have propose a new method to calculate non-linear corticomuscular coherence [94], with which they have found non-linear corticomuscular coherence during isometric wrist extension and attributed it to somatosensory feedback [95]. Such method may be extended to dynamic movements in the future.

With coherence, we also could not infer the directionality of corticomuscular communication. Although the EEG signal from C_z_ is likely to consist primarily of electrical cortical activities in the midline cortical structures such as the primary motor and sensory cortices and the supplementary motor area, the signal can also contain activities from the adjacent cortical areas through volume conduction. To determine the sources of the signal from C_z_, detailed source localization is required. However, as coherence is a linear measure, it seems more likely that coherence exists via the monosynaptic corticospinal connection rather than the polysynaptic connections for somatosensory feedback.

This study was also limited by the absence of freezing episodes among participants with PD. Such episodes could have been accompanied by observable discrepancies in corticomuscular coherence between healthy participants and participants with PD.

All our participants with PD were responsive to dopaminergic medications and were tested after overnight medication withdrawal in the practically defined off state. However, there could have been some residual effects of dopaminergic medications at the time of testing.

## Conclusions

In this study, participants with and without PD performed bilateral, anti-phasic ankle movements. Despite abnormal consistency and symmetry of movement, participants with PD did not significantly differ in the magnitude of corticomuscular coherence from participants without PD. This finding suggests that, for participants with PD, either *i*) pathological processes outside linear corticomuscular communication contributed to the kinematic abnormalities or *ii*) PD-related changes in the neural correlates of movement maintained corticomuscular communication but motor performance was still impaired. To delineate whether corticomuscular communication is involved in kinematic abnormalities in PD, future studies should also compare the neural correlates of movement between individuals with and without PD.
